# Genome survey sequencing provides clues into glucosinolate biosynthesis and flowering pathway evolution in allotetrapolyploid *Brassica juncea*

**DOI:** 10.1186/1471-2164-15-107

**Published:** 2014-02-06

**Authors:** Jinghua Yang, Ning Song, Xuan Zhao, Xiaohua Qi, Zhongyuan Hu, Mingfang Zhang

**Affiliations:** 1 Laboratory of Germplasm Innovation and Molecular Breeding, Institute of Vegetable Science, Zhejiang University, Hangzhou 310058, P. R. China; 2 Key laboratory of Horticultural Plant Growth, Development & Quality Improvement, Ministry of Agriculture, Hangzhou 310058, P. R. China; 3 College of Horticulture and Plant Protection, Yangzhou University, Yangzhou 225009, P. R. China

**Keywords:** *Brassica juncea*, Comparative genome analysis, Flowering pathway, Genome survey sequencing, Glucosinolate biosynthesis

## Abstract

**Background:**

*Brassica juncea* is an economically important vegetable crop in China, oil crop in India, condiment crop in Europe and selected for canola quality recently in Canada and Australia. *B. juncea* (2n = 36, AABB) is an allotetraploid derived from interspecific hybridization between *B. rapa* (2n = 20, AA) and *B. nigra* (2n = 16, BB), followed by spontaneous chromosome doubling.

**Results:**

Comparative genome analysis by genome survey sequence (GSS) of allopolyploid *B. juncea* with *B. rapa* was carried out based on high-throughput sequencing approaches. Over 28.35 Gb of GSS data were used for comparative analysis of *B. juncea* and *B. rapa*, producing 45.93% reads mapping to the *B. rapa* genome with a high ratio of single-end reads. Mapping data suggested more structure variation (SV) in the *B. juncea* genome than in *B. rapa*. We detected 2,921,310 single nucleotide polymorphisms (SNPs) with high heterozygosity and 113,368 SVs, including 1-3 bp Indels, between *B. juncea* and *B. rapa*. Non-synonymous polymorphisms in glucosinolate biosynthesis genes may account for differences in glucosinolate biosynthesis and glucosinolate components between *B. juncea* and *B. rapa*. Furthermore, we identified distinctive vernalization-dependent and photoperiod-dependent flowering pathways coexisting in allopolyploid *B. juncea*, suggesting contribution of these pathways to adaptation for survival during polyploidization.

**Conclusions:**

Taken together, we proposed that polyploidization has allowed for accelerated evolution of the glucosinolate biosynthesis and flowering pathways in *B. juncea* that likely permit the phenotypic variation observed in the crop.

## Background

The *Brassicaceae* family includes approximately 3,700 species in 350 genera with diverse characteristics, many of which are of agronomic importance as vegetables, condiments, fodder and oil crops [1]. The genus *Brassica* contains the majority of crop species of *Brassicaceae* family. Of particular importance are the cole crop and vegetable species *B. rapa*, *B. oleracea*, *B. napus*, and *B. juncea* as sources of oils and vegetables. Because of their agricultural importance, genome components of several *Brassica* species have been characterized in detail over the past few years [2-4]. The genomes of three diploid species, *B. rapa* (AA, 2n = 20), *B. nigra* (BB, 2n = 16), and *B. oleracea* (CC, 2n = 18), have been shown to contain triplicate homologous counterparts of corresponding segments in the *Arabidopsis* genome due to whole-genome triplication that occurred approximately 12–17 million years ago [1,5]. Additional natural allopolyploidization events in the last 10,000 years, have resulted in the creation of three allotetraploid hybrids, *B. juncea* (AABB, 2n = 36), *B. napus* (AACC, 2n = 38) and *B. carinata* (BBCC, 2n = 34) [6-10]. *B. juncea* is used as a vegetable in China and Southeastern Asia, and is a source of oil in India and Europe. The species possesses unique traits that include much wider morphological variation in leafy types, root type, stem type, seed stalk type and oil type [11]. *B. juncea* has been reported to contain higher glucosinolates than other *Brassica* species [12]. Glucosinolates are of higher value to human nutrition that may reduce the risk of cancer incidence. In addition, they are toxic to some soil-borne plant pathogens, hence, accounting for their selection [13,14].

The recent accomplishment of genome sequencing and annotation of *B. rapa*[5], combined with the available genome sequence data for model *Arabidopsis* in *Brassicaceae*[15], provide improved strategies for comparative genome analysis and breeding. Attempts to develop a unified comparative genomics system in the *Brassicaceae* have revealed 24 conserved genomic blocks [4], an extension to the 21 syntenic blocks identified in *B. napus*[16]. Comparative mapping studies between members of *Brassica* and *Arabidopsis thaliana*[16-22], and *Arabidopsis thaliana* and *Capsella rubella*[23], together with the identification of an ancestral karyotype (AK) [24], have stimulated interest in the evolutionary processes underlying diversification in the *Brassicaceae*. Since the allotetraploid species possess much larger genomes than their diploid counterparts in *Brassica*[2], we expect that novel gene/pathway interactions have emerged in the allotetraploid *Brassica* species through sub-functionalization and/or neo-functionalization of paralogs [25,26].

Low coverage genome survey sequences (GSS) can provide information about gene content, polymorphism, functional elements, repetitive elements and molecular markers [27-31]. In some studies, most of the coding sequence in a genome can be surveyed with less than 2 genome coverage [32]. It was possible to recover 38% of the coding fraction of the mouse-human alignment with only 0.66 × coverage of the pig genome [33]. With only 0.1 × coverage, it was possible to generate a considerable amount of biologically useful information and genomic resources for *Megaselia scalaris*, including identification of repetitive elements, the mitochondrial genome, microsatellites and identification of gene homologs [34]. These studies make a compelling case for low density sequencing in the genomic studies of non-model species.

Here, we employed high-throughput sequencing for comparative genome analysis of *B. juncea* and *B. rapa* to identify genome changes associated with polyploidization that might account for the phenotypic diversity of *B. juncea*. We showed clues of glucosinolate biosynthesis and flowering pathway evolution occurred in *Brassica juncea,* likely accounting for some of the phenotypic diversity that is observed. Furthermore, it provides a valuable resource for more focused investigations into the rate and distribution of genomic changes that accompany polyploidization in this species.

## Results

### *Karyotype of B. juncea*

**Figure 1:**
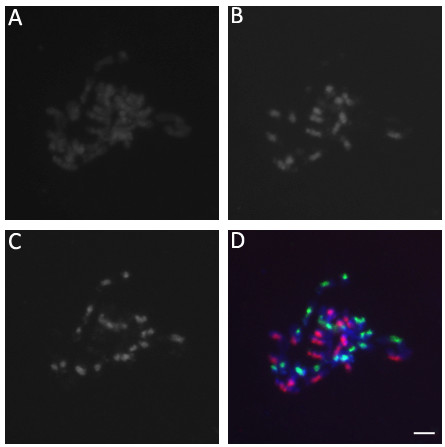
**Genomic *in situ *hybridization analysis of genome component in *B. juncea*.** Metaphase chromosome from root tip cell of *B. juncea***(A)**, detection of B genome chromosome in *B. juncea* chromosome **(B)**, detection of A genome chromosome in *B. juncea* chromosome **(C)**, A and B genomes with red and green fluorescence in *B. juncea***(D)**. Bar = 5 μm.

According to the 'U-triangle’ theory of *Brassicaceae*[6], allotetraploid *B. juncea* originated from hybridization of *B. rapa* (AA, 2n = 20) and *B. nigra* (BB, 2n = 16). We identified genomic components of *B. juncea* by genomic *in situ* hybridization (GISH). The two predicted genomes (A and B) of the allotetraploid were distinguished using genomic DNA from *B. rapa* and *B. nigra* as probes representing the putative progenitor genomes. The 20 A and 16 B chromosomes detected suggest that the two genomes have remained somewhat distinct in *B. juncea* with no significant genome homogenization and no large-scale translocations between genomes (Figure 1).

### Comparative genome analysis of *B. juncea and B. rapa*

After quality evaluation of sequencing data (Additional file 1: Figure S1), a total of 28.35 Gb high quality data were collected for the *B. juncea* genome and used to compare with whole genome sequence of *B. rapa*. It was feasible to map 45.93% sequences of the *B. juncea* GSS data to the genome sequences of *B. rapa*. Of these, only 18.44% single-end reads were mapped to the genome sequences of *B. rapa*, which indicated more SV in the *B. juncea* genome compared to *B. rapa*. The identity of mapped sequences is 98.14%, which shows a close genetic relationship between *B. juncea and B. rapa* (Additional file 1: Table S1). The coverage depth and distribution on chromosomes suggest a high comparison ratio over the *B. rapa* genome (Additional file 1: Figure S2).

Polymorphism analysis identified 2,921,310 SNPs, including 58.53% transitions, 41.47% transversions and 58.19% heterozygosity. We showed the distributions of SNP-type in 10 chromosomes of *B. rapa* genome (Additional file 1: Table S2, Additional file 1: Figure S3). 44,053 SVs were detected as insertions and deletions, with approximately even distributions of SVs across the 10 chromosomes of *B. rapa* genome (Additional file 1: Table S3, Additional file 1: Figure S3). 69,315 Indel (1–3 bp) polymorphisms were also observed, of which 1 bp-sized Indels were most abundant in genome and 3 bp-sized Indels were most abundant in coding sequence (Additional file 1: Table S4, Additional file 1: Figure S3). Most SNPs and SVs (including 1–3 bp Indels) were located in exon, intron, transposon, intergenic, TEprotein, TandemRepeat region of genome, others were found in miRNA, tRNA and snRNA coding regions of genome (Table 1). These SNPs cause a relatively high ratio of non-synonymous mutations in genes; for example, 9680 genes were found with (≧10) non-synonymous SNPs. Moreover, 1448 genes coding regions were changed by frame-shift Indels, and we also found 5989 genes have SV within gene coding regions (Table 2). A number of gene functions were found to be altered by these mutations based on Non-Redundant Nucleotide Database (NT/NR), Cluster of Othologues Groups Proteins Database (COG) and Kyoto Encyclopedia of Genes and Genomes Database (KEGG) database searches (data not shown). Here, we have focused on glucosinolate biosynthesis and flowering pathways in particular.

**Table 1:** **Distribution of SNPs and SVs polymorphisms in genomic components in *B*. *juncea***

**Type**	**Exon**	**Intron**	**miRNA**	**tRNA**	**snRNA**	**TEprotein**	**Transposon**	**TandemRepeat**	**Intergenic**
**SNP**	912059	276795	393	789	671	88942	629793	59754	952114
SV*	3769	9038	14	33	51	2374	11901	2896	13977
Indel*	3294	17253	11	21	19	2314	15313	1459	29631

SV*: >3 bp.

Indel*: 1–3 bp.

**Table 2:** **Statistics of non-synonymous mutations by SNPs, genes with Frame-shift by Indels and genes with SVs in *B*. *juncea***

**Genes with Non-synonymous SNPs: total detected SNPs**	**Genes with( > =10) Non-synonymous SNPs: total detected SNPs**	**Genes with Frame-shift Indels: total detected Indel**	**Genes with SVs: total detected SVs**
35457: 278241	9608: 166533	1448: 1528	5989: 7059

### Glucosinolate biosynthesis genes expression between *B. juncea* and *B. rapa*

**Figure 2:**
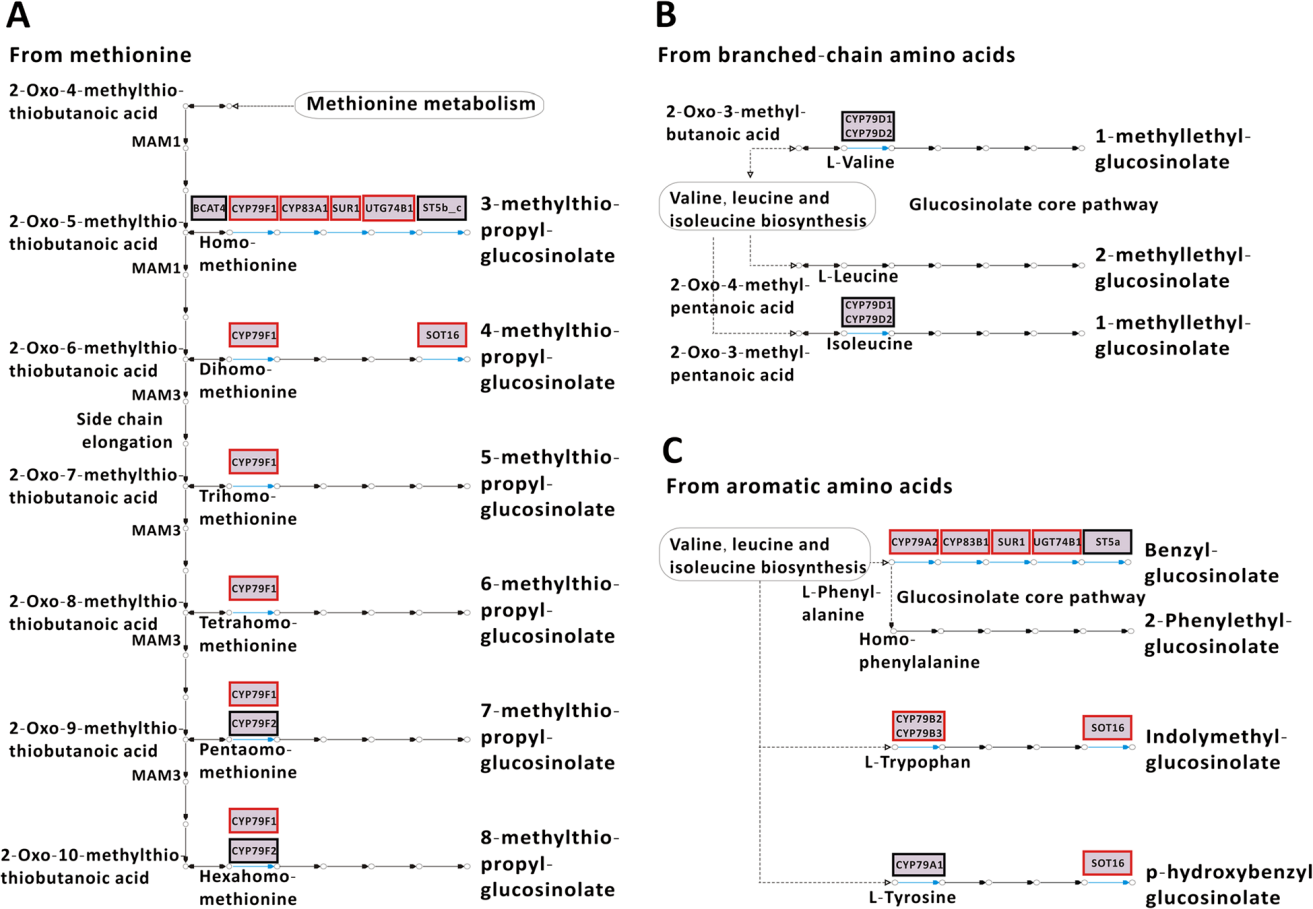
**The three glucosinolate biosynthesis pathway in *B. juncea *by KEGG analysis.** Glucosinolate biosynthesis from methionine **(A)**, glucosinolate biosynthesis from branched-chain amino acids **(B)** and glucosinolate biosynthesis from aromatic amino acid **(C)**. The red frames show polymorphic genes from the non-synonymous polymorphism compared to *B. rapa*.

**Figure 3:**
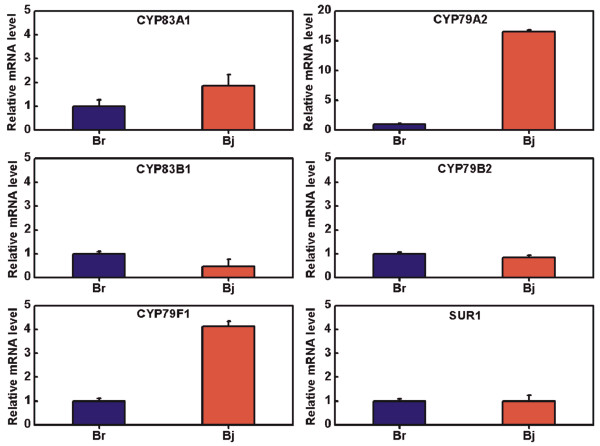
**Transcriptional patterns of glucosinolate biosynthesis related genes in *B*. *juncea *** **and *B*. *rapa*.**

We constructed glucosinolate biosynthesis pathway in *B. juncea* by KEGG analysis. Three biosynthesis pathways were identified from different substrates including methionine, branched-chain amino acid and aromatic amino acid (Figure 2). Among glucosinolate biosynthesis-related genes, we found non-synonymous SNPs and deletion/insertion SV polymorphisms in *CYP79F1* (*CYP*, *cytochromes P450*), *CYP83A1*, *SUR1* (*SUPERROOT1*), *UGT74B1* (*UDP*-*glucose*:*thiohydroximate S*-*glucosyltransferase*), *SOT16* (*sulfotransferase*), *CYP79A2*, *CYP83B1*, *CYP79B2* and *CYP79B3* genes (Additional file 1: Table S5), which suggested different genes expressions and glucosinolate components and contents. Gene expression of 6 selected glucosinolate biosynthesis-related genes were investigated in leaves between *B. juncea* and *B. rapa. CYP83A1*, *CYP79A2* and *CYP79F1* expressions were up-regulated in *B. juncea* than *B. rapa. CYP83B1* expression was down-regulated in *B. juncea* than *B. rapa*. There was no difference in *CYP79B2* and *SUR1* expressions between *B. juncea* and *B. rapa* (Figure 3). These mutations appear to cause differences in gene expression and glucosinolate content between *B. juncea* and *B. rapa*.

### Glucosinolate component and content between *B. juncea* and *B. rapa*

**Figure 4:**
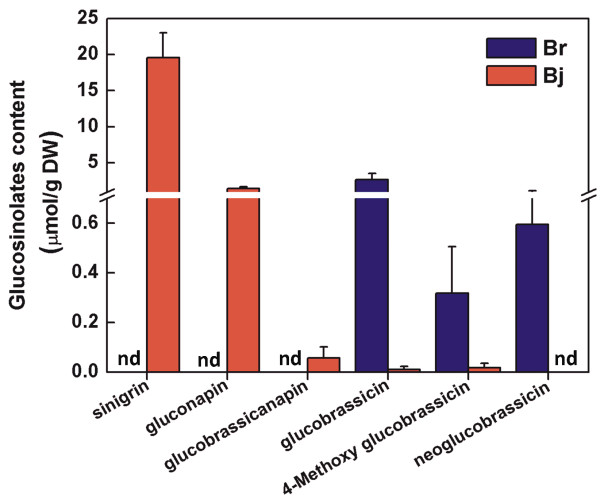
**Glucosinolates components and contents in *B*. *juncea *** **and *B*. *rapa***

We checked glucosinolate component and content between *B. juncea* and *B. rapa* by HPLC. Of glucosinolate component, sinigrin, gluconapin, glucobrassicanapin, glucobrassicin and 4-Methoxy glucobrassicin were detected in young leaves of *B. juncea*, of which sinigrin showed very high content with 19.58 μ mol/g DW in leaves. Only glucobrassicin, 4-Methoxy glucobrassicin and neoglucobrassicin were detected in young leaves of *B. rapa* (Figure 4).

### The flowering pathway in *Brassica juncea*

**Figure 5:**
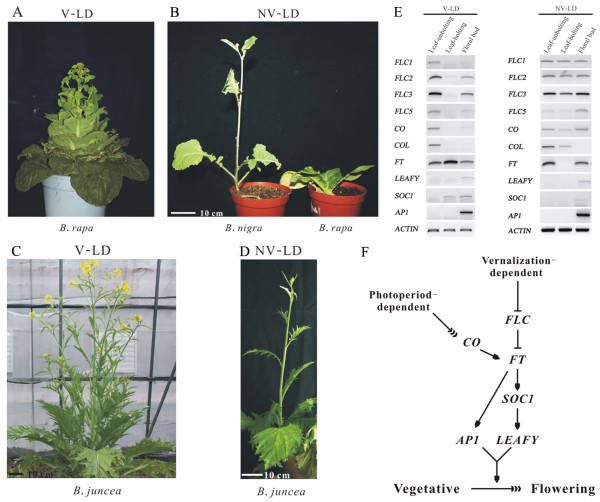
**Vernalization- and photoperiod-dependent flowering pathways coexist in allotetraploid *B. juncea*.** Vernalization and long-day photoperiod condition (**A** and **C**), Non-vernalization and long-day photoperiod condition (**B** and **D**), *B. juncea* flowering pathway related gene expression **(E)** and proposed flowering pathway in *B. juncea***(F)**

Flowering behavior is an essential feature affecting *Brassicaecae* crop production. For *B. rapa* (AA genome), seed vernalization and long-day photoperiod conditions are necessary for flowering (Figure 5-A, B), while only long-day photoperiod conditions promote *B. nigra* flowering, without any need for vernalization treatment (Figure 5-A, B). Interestingly, long-day photoperiod conditions lead to flowering in *B. juncea* regardless of vernalization conditions (Figure 5-C, D). We identified four *FLOWERING LOCI C* (*FLC1*, *FLC2*, *FLC3* and *FLC5*) genes and other flowering pathway-related genes, including *CONSTANS* (*CO*), *CONSTANS* -like (*COL*), *FLOWERING T* (*FT*), *LEAFY*, *SOC1* (*SUPPRESSOR OF OVEREXPRESSION OF CO1*) and *AP1* (*APETALA1*)*,* in *B. juncea*. Under vernalization and long-day photoperiod conditions, when *FLCs* gene expression is down-regulated, flowering occurs by an FLC-dependent pathway in *B. juncea*. Under non-vernalization and long-day photoperiod conditions, flowering occurs by a CONSTANS-dependent pathway, not FLC-dependent, since *FLCs* genes are still expressed during flowering (Figure 5-E). These results indicate that vernalization- and photoperiod-dependent flowering pathways coexist in the allotetraploid *B. juncea* (Figure 5-F).

## Discussions

Allotetraploid *B. juncea* possesses unique traits that influence its utility as a vegetable crop in China and oil crop in India; these features emerged after natural hybridization between *B. rapa* and *B. nigra* and allopolyploidization. The A genome (*B. rapa*) [5] and C genome [35] sequences were recently completed, providing considerable momentum in molecular genetic studies of *Brassica. Brassica* A/B/C genome phylogeny and evolution is of considerable interest. Largely because of the vast phenotypic diversity available within the *Brassicas*[1,6].

The fate of duplicated genes can be defined as sub-functionalization, neo-functionalization or non-functionalized after polyploidization or whole genome duplication (WGD) in polyploid crops [36,37]. Biased gene expression between homologous gene is usually observed in allopolyploid plants, including *Gossypium*[38], *Arabidopsis*[39] and *Tragopogon*[40], resulting from genetic and epigenetic interactions between redundant genes, and these interactions can influence plant phenotypes and evolutionary fates of polyploid types [37]. Among the many models that attempt to explain how/why duplicated genes are retained after polyploidy [41], sub-functionalization is the most popular hypothesis even though it remains controversial [42]. Genome plasticity, redundancy and diversity are well described and discussed in polyploid *Brassicaceae*[43-45], and are thought to contribute to adaptive phenotypic variation [37,40,46]. For example, flowering time variation is affected by the replicated copies of the flowering time gene *FLC* in *Brassicaceae*[46]. Here, we preliminarily show that vernalization-dependent and photoperiod-dependent flowering pathways coexist in allopolyploid *B. juncea,* suggesting that the flowering pathways of *B. rapa* and *B. nigra* can express in independent vernalization environments in the allopolyploid *B. juncea*. Timing of flowering onset is an essential trait that affects crop production and plant life cycle. To meet the challenges of climate changes and adapt to a wider range of growing environments, plants adjust their flowering time or pathway during evolution. The coexistence of vernalization-dependent and photoperiod-dependent flowering pathways might indicate better adaptation for survival during evolution in *B. juncea*. On the other hand, with global warming, *B. juncea* may have more potential to be used as oil crops because of its flowering trait independent of vernalization status.

In this study, we employed high-throughput sequencing approach based on Illumina/Solexa platform to investigate 30 × genome survey sequences of *B. juncea*. After comparison to *B. rapa* genome, 45.93% genome survey sequences of *B. juncea* can be mapped to *B. rapa* genome, which indicate relative far phylogenetic relationship between A/B than A/C. This provides an opportunity that we can sequence this genome by diploid approaches. After comparative genome analysis between *B. juncea* and *B. rapa*, we find more SV in *B. juncea* genome, which may be resulted from polyploidy event. Moreover, based on the 30 × genome survey sequences of *B. juncea*, we observed huge polymorphisms between *B. juncea* and *B. rapa* including SNPs, SVs and Indels. The non-synonymous SNPs, frame-shift Indels and genes with SVs resulted from these polymorphisms caused a large number of pathways to be changed in *B. juncea* by KEGG analysis, for example, glucosinolate biosynthesis pathway. Higher expressions of *CYP83A1* and *CYP79F1* genes are associated with a higher content of aliphatic glucosinolate in *B. juncea* than *B.rapa*. Increased *CYP83B1* gene expression is associated with a higher content of indole glucosinolate in *B.rapa* than *B. juncea*. However, we did not observe a higher content of aromatic glucosinolate resulting from observed higher expression of *CYP79A2* in *B. juncea* than *B. rapa*. That may be reason that we did not observe higher expressions of *SUR1* downstream of *CYP79A2* and *CYP79B2* in aromatic glucosinolate biosynthesis pathway in *B. juncea*. The advent of high-throughput sequencing (Next-generation sequencing, NGS) has revolutionized genomic and transcriptomic approaches to biology. These new sequencing tools are also valuable for discovering, sequencing and genotyping not only hundreds but thousands of markers across almost any genome of interest, even in species in which little or no genetic information is available [47].

## Conclusions

In this study, we find the clues of glucosinolate biosynthesis and flowering pathways evolution in *B. juncea* based on comparative analysis between 30 × genome survey sequences of *B. juncea* and genome of *B. rapa*, which allow us to propose that polyploidization resulted in the evolution of glucosinolate biosynthesis and flowering pathways in *B. juncea*. The genome survey sequencings promote the whole genome sequencing processing in *B. juncea*. To conclude, next-generation sequencing, even low genome coverage is pushing forward the molecular genetics especially in non-model plant.

## Methods

### Plant materials

The inbred line of *Brassica jucnea* var *tumida* Tsen et Lee from our lab (Institute of Vegetable Science, Zhejiang University) was used to conduct genome survey sequencing in this study. *Brassica rapa* and *Brassica nigra* seeds were procured from the University of Warwich and Beijing Academy of Agriculture and Forestry Sciences, respectively.

### *Genome in situ hybridization of chromosome in B. juncea*

Seeds of *B. juncea*, *B. rapa* and *B. nigra* were germinated at 28°C in dark. Root tips were harvested, in ice-bath for 24 hours and fixed in solution (Ethanol: Acetic acid = 3: 1) for 24 hours. The root tips were stained within 1% acetocarmine for 15 min and dropped on slide with 45% acetic acid then covered with a coverslip. The slides with samples were examined by microscope to find the metaphase stage of chromosome and then conserved. Total genomic DNA was isolated from young leaf tissue of *B. rapa* and *B. nigra* using a DNA extraction kit (QIAGEN, USA). The genomic DNA of *B. rapa* was labeled with biotin-16-dUTP by nick translation and the genomic DNA of *B. nigra* was labeled with digoxingenin-11-dUTP by nick translation (Roche, USA). For genomic *in situ* hybridization, slide pretreatment, chromosome denaturation with probe, hybridization and post-hybridization treatments were referred to the method [48]. The images were captured and analyzed using Zeiss Axioskop fluorescence microscope system (ZEISS, Germany).

### Library construction, sequencing and re-sequencing

Genomic DNAs were isolated from young leaf tissue of *B. juncea* using a DNA extraction kit (Illunima, USA). Genomic Paired-end libraries with 170 bp and 500 bp insertion were constructed following a standard protocol provided by Illumina. The adapter ligation and DNA cluster preparation were performed and subjected to sequencing using Illumina Genome Analyzer (Illumina Hiseq2000, USA) according to the manufacturer’s standard protocol. Low-quality reads, reads with adaptor sequences and duplicated reads were filtered, and remaining high-quality data was used in the following assembly and analysis.

### Comparative genome analysis

Genome sequence of *B. rapa* was used as reference to comparatively analyze the genome survey sequences (GSS) of *B. juncea* by using Burrows-Wheeler Aligner (BWA) program. Samtools, Pindel and Breakdancer software were used to analyze the molecular polymorphisms including SNP, SV and Indel polymorphisms by comparison of the survey genome of *B. juncea* and genome of *B. rapa*. BLAST software was used for gene annotation.

### Glucosinolate biosynthesis gene expression

Total RNA was extracted from seedlings using an RNeasy Plant Mini Kit (QIAGEN, USA) following the manufacturer’s protocol. During extraction, total RNA was exhaustively treated with RNase-free Dnase (Qiagen, Germany). RNA concentration and quality were determined with a biophotometer (Eppendorf, Germany) and gel analysis. 1 μg total RNA was transcribed to synthesize cDNA first strand using a Reverse Transcriptase M-MLV Kit (Takara, Japan). The expression of 6 selected genes was assayed in *B. juncea* and *B. rapa* by quantitative real-time PCR (qPCR) on ABI Step One (Applied Biosystems, USA). qPCR reaction were performed using 2.5 μl cDNA template, 6.5 μl of Fast start universal SYBR Green Master (Roche Germany), and 2.0 μM primer, in a total 20 μl reaction system. The relative quantification of the target gene was determined using the ^ΔΔ^CT method. All PCR reactions were run in triplicate on each plate as technical replicates and three independent biological replicates were used. Gene fragment of *CYP83A1*, *CYP79A2*, *CYP83B1*, *CYP79B2*, *CYP79F1*, *SUR1* and *25S* were cloned from *B. juncea* and *B. rapa* and conserved sequences of these genes were used for primer design. 25S was used as an internal control gene to evaluate relative gene expression level. Primers used in this study are listed in Additional file 1: Table S6.

### Glucosinolate content measurement

Duplicates of the freeze-dried powder (0.25 g) in 10 ml glass tubes were preheated for 5 min in 75°C water bath. And 4 ml of 70% boiling methanol (75°C) were added and extracted at 75°C in a water bath for 10 min. For internal standardization 100 μl of 5 mM sinigrin (Sigma-Aldrich Co., MO, USA) were added to one of the duplicates before extraction. Then 1 ml of 0.4 M barium acetate were rapidly added and the vials vortexed for several seconds. After centrifugation at 4,000 rpm for 10 min at room temperature, the supernatants were collected and the pellets were re-extracted twice with 3 ml of 70% boiling methanol (75°C). Three supernatants were combined and made up to a final volume of 10 ml with 70% methanol. 5 ml extracts were loaded onto a 1 ml mini-column (JT Baker, USA) containing 500 μl of activated DEAE Sephadex™ A-25 (Amersham Biosciences, Sweden), and allowed to desulphate overnight with aryl sulfatase (Sigma-Aldrich Co., MO, USA). The resultant desulpho (ds)-GS were eluted with 2.5 ml of ultra pure water produced by Milli-Q system (Millipore Co., USA) and stored at -20°C prior to separation by high performance liquid chromatography (HPLC).

Samples of 40 μl were analyzed in a Shimadzu HPLC system (LC-10AT pump, CTO-10A column oven, SCL-10A VP system controller, Shimadzu, Kyoto, Japan) consisting of a UV–VIS detector (SPD-10A) set at 229 nm and a prontosil ODS2 column (250 × 4 μm, 5 μm, Bischoff, Germany). The mobile phase consisted of ultrapure water (A) and acetonitrile (Tedia, USA) (B). The mobile phase was in the following gradient: H_2_O (2 min), a linear gradient of 0-20% acetonitrile (32 min), 20% acetonitrile (6 min), followed by 100% acetonitrile and 0% acetonitrile prior to the injection of the next sample.

### Identification of flowering pathway in B. juncea

For vernalization and long-day treatment, *B. juncea* and *B. rapa* were grown in glass greenhouse during winter season starting 4th week of October. Under these conditions, *B. rapa* began flowering in March and *B. juncea* in April. For non-vernalization and long-day treatment, *B. juncea*, *B. nigra* and *B. rapa* were grown in a growth chamber under conditions of 25°C and photoperiod of 16 light: 8 dark. Semi-RT-PCR method was employed to study the flowering pathway-related gene expression, including *FLC1/2/3/5*, *CO*, *COL*, *FT*, *LEAFY*, *SOC1*, *AP1. ACTIN* gene from *B. juncea* was used as an internal control gene to evaluate relative gene expression level. Degenerate primers of flowering pathway-related genes were referred to publications [49-51], NCBI Accessions JQ314107, JN699544 and cloned gene fragment. Primers of *ACTIN* gene was designed by NCBI Accessions HM565958. Primers used in this study are listed in Supporting Information Additional file 1: Table S6.

## Competing interests

The authors have declared that no competing interests exist.

## Authors’ contributions

JY and MZ conceived and designed the experiments. JY, NS, XZ, XQ and ZH performed the experiments and data analysis. JY wrote the paper and MZ edited the paper. All authors read and approved the final manuscript.

## Supplementary Material

Additional file 1: Table S1 Statistical comparison of sequencing reads of *B. juncea* with genome of *B. rapa*. **Table S2:** Statistic of SNPs between mapped sequences of *B. juncea* and *B. rapa* genome sequence. **Table S3:** Statistic of SVs between mapped sequences of *B. juncea* and *B. rapa* genome sequence. **Table S4:** Statistic of Indel (1–3 bp) between mapped sequences of *B. juncea* and *B. rapa* genome sequence. **Table S5:** Polymorphism information on glucosinolate biosynthesis related genes between *B. juncea* and *B. rapa.***Table S6:** Primer sequences used for qRT-PCR and RT-PCR. **Figure S1:** Estimation of high-throughput sequencing quality including insert size, quality distribution, nucleotide content and cycle average quality distribution. **Figure S2:** Comparison of in-depth distribution of sequencing reads from *B. juncea* on chromosome of *B. rapa*. **Figure S3:** Distribution of SNP and SV polymorphisms on chromosome of *B. rapa*. **Figure S4:** Distribution of 1–3 bp Indels in genome and coding sequence (CDS) region of *B. rapa*. Click here for file
