# Supplementary material for: Genome survey sequencing provides clues into glucosinolate biosynthesis and flowering pathway evolution in allotetrapolyploid Brassica juncea
Source: BMC Genomics. 2014 Feb 6;15:107. doi: 10.1186/1471-2164-15-107 (PMC3925957; doi:10.1186/1471-2164-15-107)
Supplement: Additional file 1: Table S1 — Statistical comparison of sequencing reads of B. juncea with genome of B. rapa. Table S2: Statistic of SNPs between mapped sequences of B. juncea and B. rapa genome sequence. Table S3: Statistic of SVs between mapped sequences of B. juncea and B. rapa genome sequence. Table S4: Statistic of Indel (1–3 bp) between mapped sequences of B. juncea and B. rapa genome sequence. Table S5: Polymorphism information on glucosinolate biosynthesis related genes between B. juncea and B. rapa.Table S6: Primer sequences used for qRT-PCR and RT-PCR. Figure S1: Estimation of high-throughput sequencing quality including insert size, quality distribution, nucleotide content and cycle average quality distribution. Figure S2: Comparison of in-depth distribution of sequencing reads from B. juncea on chromosome of B. rapa. Figure S3: Distribution of SNP and SV polymorphisms on chromosome of B. rapa. Figure S4: Distribution of 1–3 bp Indels in genome and coding sequence (CDS) region of B. rapa. [file 1471-2164-15-107-S1.doc]

Table S1 Statistics comparison of sequencing reads of *B. juncea* with genome of *B. rapa*

| Total reads | PE (%) | SE (%) | Total map (%) | Identity (%) | depth | Cover_ratio (%) |
| --- | --- | --- | --- | --- | --- | --- |
| 304,440,194 | 27.49 | 18.44 | 45.93 | 98.14 | 40.87 | 92.28 |

*PE: pair-end reads

*SE: single-end reads

Table S2 Statistics of SNPs between mapped sequences of *B. juncea* and *B. rapa* genome sequence

| **Chr** | **SNP number** | **Transition (%)** | **Transversion (%)** | **Heterozygosity (%)** |
| --- | --- | --- | --- | --- |
| **A01** | 320850 | 58.74 | 41.26 | 57.15 |
| **A02** | 297320 | 58.45 | 41.55 | 56.19 |
| **A03** | 348842 | 58.29 | 41.71 | 60.41 |
| **A04** | 196068 | 58.56 | 41.44 | 56.59 |
| **A05** | 235514 | 58.44 | 41.56 | 63.16 |
| **A06** | 281393 | 58.56 | 41.44 | 60.61 |
| **A07** | 255739 | 58.65 | 41.35 | 57 |
| **A08** | 234317 | 58.37 | 41.63 | 56.22 |
| **A09** | 403949 | 58.62 | 41.38 | 55.93 |
| **A10** | 202346 | 58.6 | 41.4 | 59.62 |
| **total** | 2776338 | 58.53 | 41.47 | 58.19 |

*Chr: Chromosome

*A01 – A10: chromosome number of *B*. *rapa*

Table S3 Statistics of SVs between mapped sequences of *B. juncea* and *B. rapa* genome sequence

| **Chr** | **SV** | **INS (%)** | **DEL (%)** | **IDE (%)** | **INV (%)** | **ITX (%)** | **CTX (%)** |
| --- | --- | --- | --- | --- | --- | --- | --- |
| **A01** | 10406 | 50.26 | 46.94 | 0 | 0.17 | 0.29 | 2.34 |
| **A02** | 11692 | 48.4 | 46.59 | 0 | 0.11 | 0.35 | 4.55 |
| **A03** | 8024 | 49.1 | 47.22 | 0 | 0.09 | 0.42 | 3.17 |
| **A04** | 11191 | 48.84 | 46.39 | 0 | 0.08 | 0.33 | 4.35 |
| **A05** | 6966 | 49.67 | 46.3 | 0 | 0.22 | 0.36 | 3.46 |
| **A06** | 7289 | 51.3 | 48.37 | 0 | 0.16 | 0.16 | 0 |
| **A07** | 8034 | 49.6 | 48.53 | 0 | 0.14 | 0.32 | 1.41 |
| **A08** | 9596 | 50.05 | 48.06 | 0 | 0.08 | 0.2 | 1.6 |
| **A09** | 13398 | 49.13 | 46.92 | 0 | 0.14 | 0.26 | 3.55 |
| **A10** | 14401 | 50.28 | 49.01 | 0 | 0.1 | 0.28 | 0.32 |
| **Total** | 100997 | 49.61 | 47.45 | 0 | 0.13 | 0.3 | 2.52 |

*Chr: Chromosome

*A01 – A10: chromosome number of *B*. *rapa*

*SV: Structure Variation; INS: Insertion; DEL: Deletion; IDE: Deletion and Insertion; INV: Inversion; ITX: intra-chromosomal translocation; CTX: inter-chromosomal translocation

Table S4 Statistics of Indel (1-3 bp) between mapped sequences of *B. juncea* and *B. rapa* genome sequence

| **Chr** | **Number** | **DEL (%)** | **INS (%)** |
| --- | --- | --- | --- |
| **A01** | 7197 | 49.48 | 50.52 |
| **A02** | 6798 | 48.25 | 51.75 |
| **A03** | 8201 | 48.38 | 51.62 |
| **A04** | 4239 | 47.32 | 52.68 |
| **A05** | 4960 | 49.42 | 50.58 |
| **A06** | 6449 | 47.91 | 52.09 |
| **A07** | 6021 | 48.88 | 51.12 |
| **A08** | 5048 | 49.37 | 50.63 |
| **A09** | 9197 | 48.33 | 51.67 |
| **A10** | 4658 | 47.66 | 52.34 |
| **Total** | 62768 | 48.52 | 51.48 |

*Chr: Chromosome

*A01 – A10: chromosome number of *B*. *rapa*

*INS: Insertion; DEL: Deletion;

Table S5 Polymorphism information on glucosinolate biosynthesis related genes between *B. juncea* and *B. rapa*

| **Gene** | **Gene ID in *B*. *rapa*** | **Polymorphism type** | **Position in gene element** |
| --- | --- | --- | --- |
| *CYP79F1* | Bra005400 | S/NS | exon |
|  |  | DEL/INS | intron |
|  | Bra032926 | S/NS | exon |
|  | Bra016907 | S/NS | exon |
|  |  | DEL | intron |
|  | Bra026058 | S/NS | exon |
|  | Bra02904 | S/NS | exon |
|  |  | DEL/INS | intron |
| *CYP83A1* | Bra016908  Bra032734 | S/NS  S/NS | Exon  Exon |
| *SUR1* | Bra024204  Bra036490 | S/NS  S/NS | Exon  Exon |
| *UGT74B1* | Bra024634 | S/NS | Exon |
| *SOT16* | Bra003819  Bra008132  Bra015935 | S/NS  S/NS  S/NS | Exon  Exon  Exon |
| *CYP79A2* | Bra009100  Bra028764  Bra040154 | S/NS  S/NS  S/NS | Exon  Exon  Exon |
| *CYP83B1* | Bra034941 | S/NS | Exon |
| *CYP79B2* | Bra011821  Bra017871 | S/NS  S/NS | Exon  Exon |
| *CYP79B3* | Bra030246 | S/NS | Exon |

S: SNP

NS: non-synonymous

DEL: deletion

INS: insertion

Table S6 Primer sequences used for qRT-PCR and RT-PCR

| Gene | Forward primer | Reverse primer |
| --- | --- | --- |
| *CYP83A1* | GAGAATGTGCCCTGGAATG | ATTCCGTTGGGAAGTTTGAA |
| *CYP79A2* | GAGGCTCACCCTATTCCTGA | CCGGAGCATTTAGTCACGTA |
| *CYP83B1* | GGCCATGACAGAACTCGTTA | CCTCTCCTTGTTGAGCCCTA |
| *CYP79B2* | ACGAGATAAACCGGAGATCG | CGGAGAGACATCTCAACGAA |
| *CYP79F1* | CACCTCGAAGGAGATGGAAT | GTACCAAACGACACGAAACG |
| *SUR1* | GACCAAACGCAAACATCTTG | AGAAGATCGAACTTGCGGAT |
| *25S* | CGGTTCCTCTCGTACTAGGTTGA | CCGTCGTGAGACAGGTTAGTTTT |
| *FLC1* | CTTGAGGAATCAAATGTCGATAA | CCATCTGGCTAGCCAAAACAT |
| *FLC2* | AACATGCTGATGATCTTAAGGCTC | CCCTGGTTCTCTTCTTTCAGCATT |
| *FLC3* | GTGGAATCAAATGTCGGTGG | AGCCAAAGCCTGATTCTCTTC |
| *FLC5* | CCTCGTTGAGCTAGAAGATCA | GGAGATTTGTCCAGATGACATCTCT |
| *CO* | TCATGTGAGCGWGCCCC | GAAGCATACCTTATYGTCTTCTC |
| *COL* | AGAAGATGAAGCAGAGGC | ACTGTAATCGACAAGGTCCAG |
| *FT* | GTGTTACGAAAATCCAAG | AAGAAGCCATCTAAAGTCT |
| *LEAFY* | GACGAACCAAGTRTTCAGGTACGC | CAGCTTTGCCATTTGGAGCG |
| *SOC1* | TCAGATGATGCGAATAGA | AGAACAAGGTAACCCAAT |
| *AP1* | TGAAGTTGCCCTTGTTGT | TCCTTAGTTCCAGCGTTT |
| *ACTIN* | CGCCGAGCGGGAAATCGTC | GGAAAGTGCTGAGGGATGC |


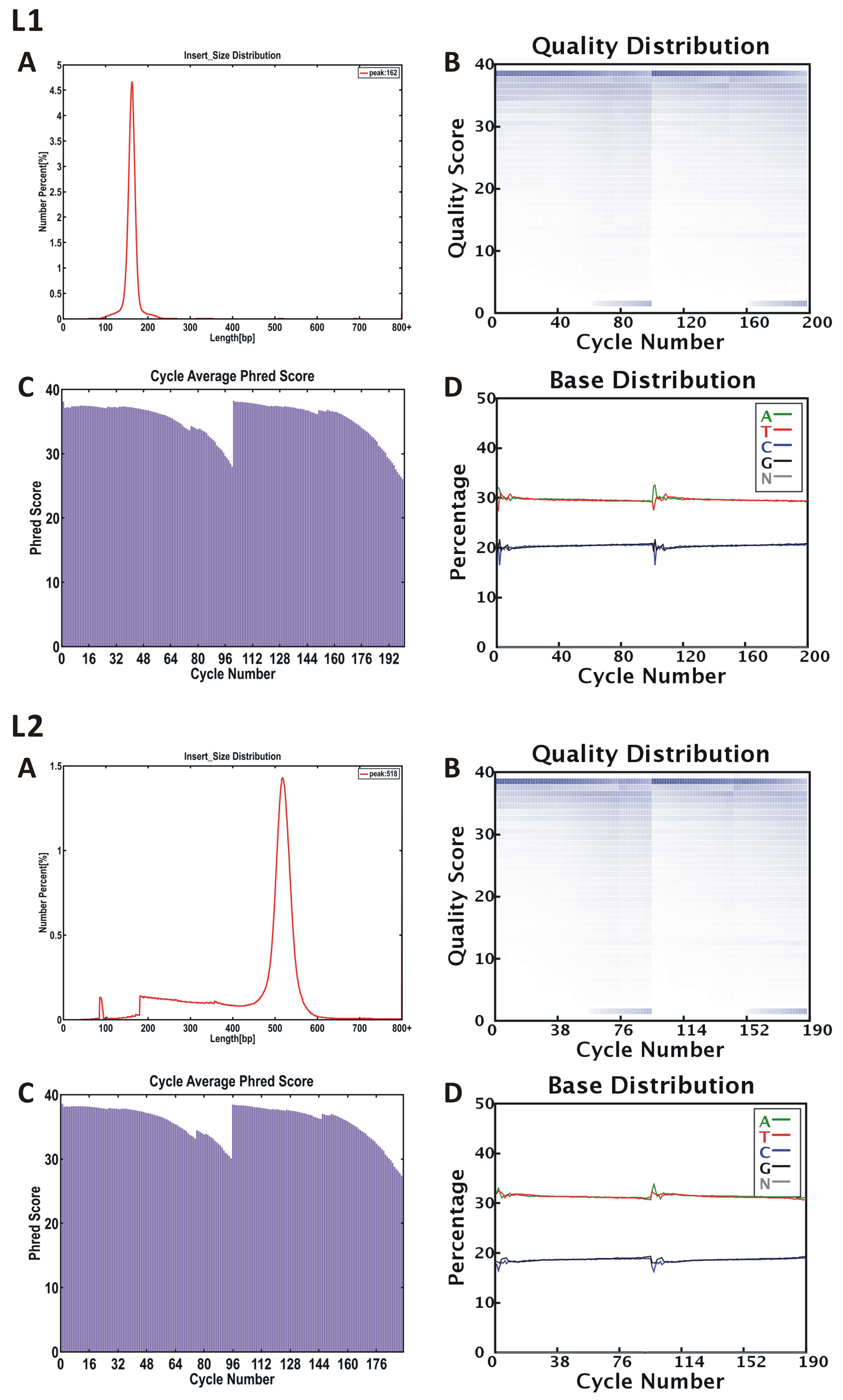


Figure S1


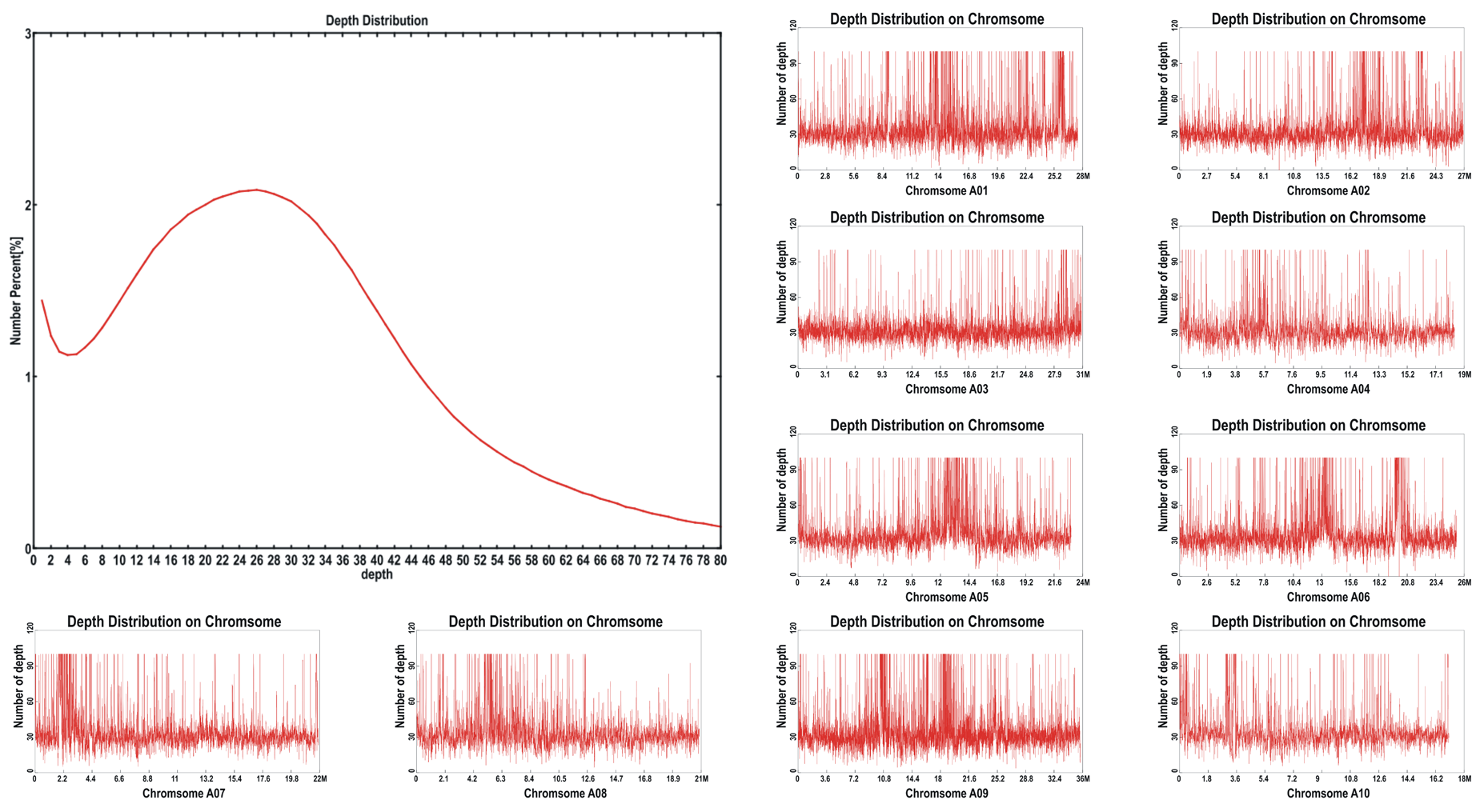


Figure S2


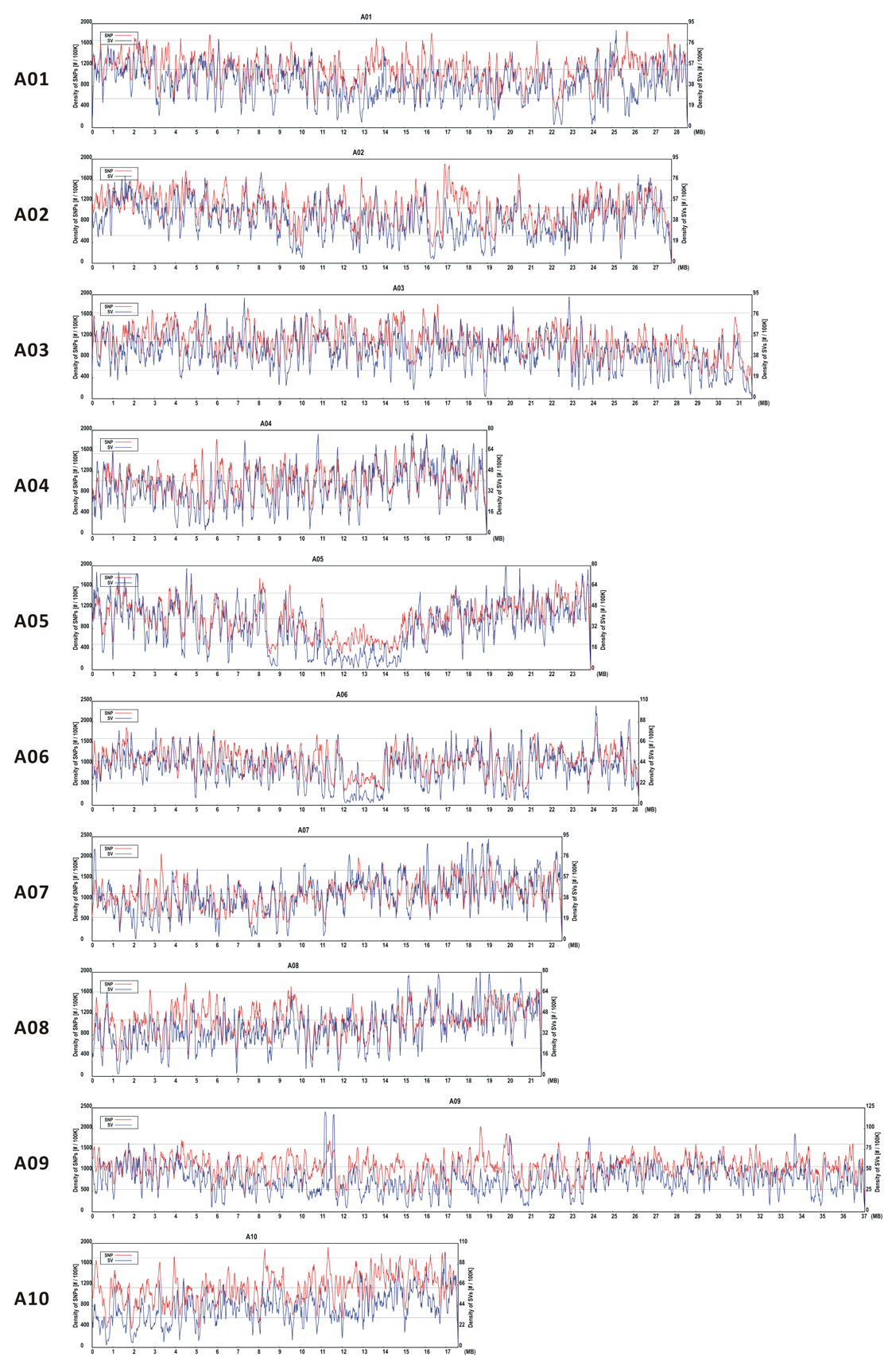


Figure S3


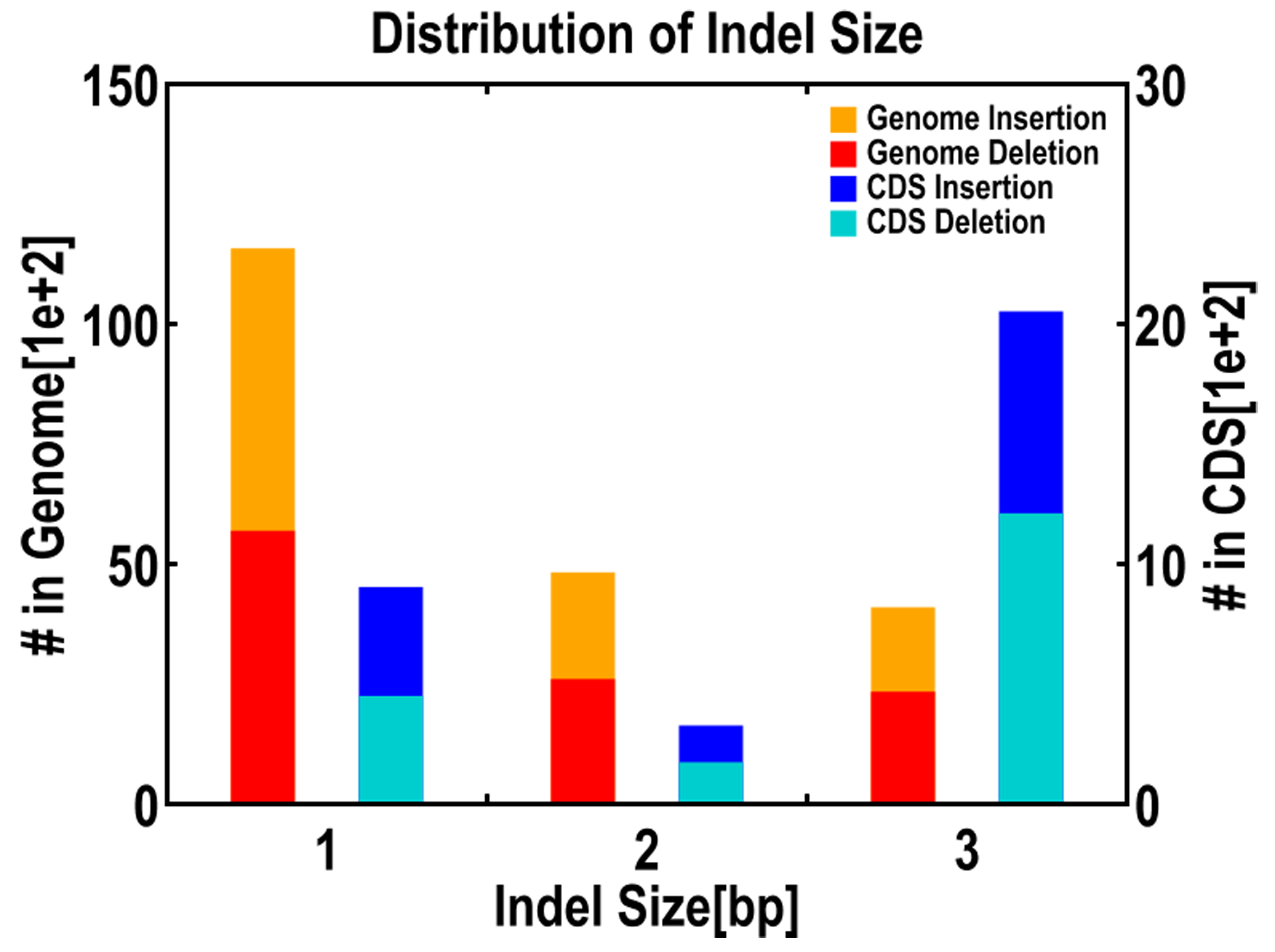


Figure S4
